# Structural plasticity of the coiled–coil interactions in human SFPQ

**DOI:** 10.1093/nar/gkae1198

**Published:** 2024-12-19

**Authors:** Heidar J Koning, Jia Y Lai, Andrew C Marshall, Elke Stroeher, Gavin Monahan, Anuradha Pullakhandam, Gavin J Knott, Timothy M Ryan, Archa H Fox, Andrew Whitten, Mihwa Lee, Charles S Bond

**Affiliations:** School of Molecular Sciences, The University of Western Australia, 35 Stirling Highway, Crawley, Western Australia 6009, Australia; School of Molecular Sciences, The University of Western Australia, 35 Stirling Highway, Crawley, Western Australia 6009, Australia; School of Molecular Sciences, The University of Western Australia, 35 Stirling Highway, Crawley, Western Australia 6009, Australia; WA Proteomics Facility, School of Molecular Sciences, University of Western Australia, Perth, WA 6009, Australia; Harry Perkins Institute of Medical Research, 6 Verdun Street, Nedlands WA 6009, Australia; School of Molecular Sciences, The University of Western Australia, 35 Stirling Highway, Crawley, Western Australia 6009, Australia; Monash Biomedicine Discovery Institute, Department of Biochemistry & Molecular Biology, Monash University, Clayton, Victoria 3800, Australia; Australian Synchrotron, 800 Blackburn Road, Clayton, VIC 3168, Australia; School of Human Sciences, The University of Western Australia, 35 Stirling Highway, Crawley, Western Australia 6009, Australia; ANSTO New Illawarra Rd, Lucas Heights, NSW 2234, Australia; School of Chemistry and Bio21 Molecular Science and Biotechnology Institute, University of Melbourne, Parkville, Victoria 3010, Australia; School of Molecular Sciences, The University of Western Australia, 35 Stirling Highway, Crawley, Western Australia 6009, Australia

## Abstract

The proteins SFPQ (splicing Factor Proline/Glutamine rich) and NONO (non-POU domain-containing octamer-binding protein) are mammalian members of the Drosophila Behaviour/Human Splicing (DBHS) protein family, which share 76% sequence identity in their conserved 320 amino acid DBHS domain. SFPQ and NONO are involved in all steps of post-transcriptional regulation and are primarily located in mammalian paraspeckles: liquid phase-separated, ribonucleoprotein sub-nuclear bodies templated by NEAT1 long non-coding RNA. A combination of structured and low-complexity regions provide polyvalent interaction interfaces that facilitate homo- and heterodimerisation, polymerisation, interactions with oligonucleotides, mRNA, long non-coding RNA, and liquid phase-separation, all of which have been implicated in cellular homeostasis and neurological diseases including neuroblastoma. The strength and competition of these interaction modes define the ability of DBHS proteins to dissociate from paraspeckles to fulfil functional roles throughout the nucleus or the cytoplasm. In this study, we define and dissect the coiled–coil interactions which promote the polymerisation of DBHS proteins, using a crystal structure of an SFPQ/NONO heterodimer which reveals a flexible coiled–coil interaction interface which differs from previous studies. We support this through extensive solution small-angle X-ray scattering experiments using a panel of SFPQ/NONO heterodimer variants which are capable of tetramerisation to varying extents. The QM mutant displayed a negligible amount of tetramerisation (quadruple loss of function coiled-coil mutant L535A/L539A/L546A/M549A), the Charged Single Alpha Helix (ΔCSAH) variant displayed a dimer-tetramer equilibrium interaction, and the disulfide-forming variant displayed constitutive tetramerisation (R542C which mimics the pathological *Drosophila nonA^diss^* allele). We demonstrate that newly characterised coiled–coil interfaces play a role in the polymerisation of DBHS proteins in addition to the previously described canonical coiled–coil interface. The detail of these interactions provides insight into a process critical for the assembly of paraspeckles as well as the behaviour of SFPQ as a transcription factor, and general multipurpose auxiliary protein with functions essential to mammalian life. Our understanding of the coiled coil behaviour of SFPQ also enhances the explanatory power of mutations (often disease-associated) observed in the DBHS family, potentially allowing for the development of future medical options such as targeted gene therapy.

## Graphical Abstract

**Graphical Abstract:**
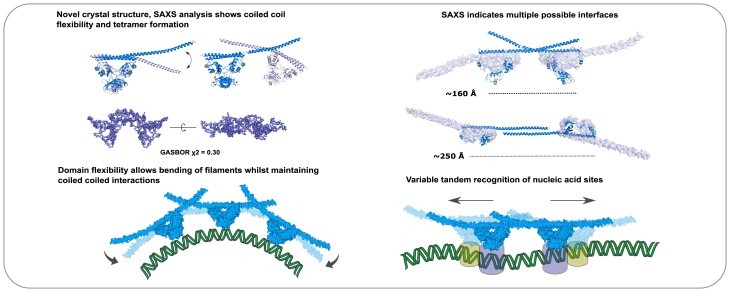

## Introduction

SFPQ, NONO and PSPC1 are the mammalian paralogues of the Drosophila Behaviour/Human Splicing (DBHS) protein family. These ubiquitous RNA binding proteins have many roles in nucleic-acid processing pathways, including transcription, splicing, RNA transport and in the formation of nuclear condensates termed paraspeckles (1). DBHS proteins enact these varied roles through a striking structural arrangement of nucleic acid binding, dimerisation and polymerisation domains, as well as extensive low complexity regions, the latter of which have proven roles in liquid phase separation (1–3). The functional importance of DBHS proteins to cell health and disease is also extensive. For example, SFPQ has a critical role in the development and regulation of neurons such that the imbalanced nucleocytoplasmic distribution of SFPQ is an important factor in the neurodegenerative diseases ALS, FTLD and AD (4,5). In the context of cancer, DBHS proteins can form oncogenic gene fusions, exhibit extensive mutations in different cancer subtypes, with effects on misregulation of transcription, splicing and epigenetic regulation (6).

DBHS proteins contain a 320 amino acid conserved, structured, core region (the DBHS region) and extensive flanking low complexity regions which vary substantially between paralogues. The DBHS core is responsible for directing homo- and heterodimerisation, and this has been well-studied with crystal structures of all six permutations having been determined and analysed, ((7) (PDB code: 5WPA); (8) (PDB codes: 5IFN, 5IFM); (9) (PDB codes: 4WII, 4WIK, 4WIJ); (10) (PDB code: 3SDE); (11) (PDB code: 7LRQ); (12) (PDB code: 7PU5)) and in many cases complemented with solution scattering studies. Previous evidence indicates that most biological functions of SFPQ and NONO are almost always performed by SFPQ/NONO heterodimers (7) making them arguably the most physiologically relevant DBHS dimer configuration. Nevertheless, the direct involvement of the DBHS region in nucleic acid interaction (8,9,13,14) suggests a potential biological role for this combinatorial expansion.

While the low complexity regions are implicated in liquid-liquid phase separation (3,15) an intermediate part of the DBHS conserved region is responsible for functional aggregation via a highly conserved antiparallel coiled-coil-forming interface, which plays a role in the cooperative binding of larger nucleic acids (9). A quadruple mutant (QM) of key hydrophobic coil-forming residues abrogated this interaction with functional consequences for gene regulation in cells. Notably, previous studies on a mutant allele of nonA – a DBHS ortholog from *Drosophila* (16) – associated an R548C mutation also present in the coiled–coil domain with an aberrant neurological phenotype. R548 is conserved as R542 in human SFPQ, which sits at the symmetry axis of the antiparallel coiled-coil in SFPQ structures (with a Cβ -Cβ distance of 6.5 Å between R542 and R542’) allowing formation of a disulfide bond in oxidising conditions. Hypothesising that the aberrant phenotype observed by Rendahl *et al.* (16) is most likely the result of constitutive polymerisation of nonA, we made the equivalent R542C substitution in SFPQ to investigate its effect *in vitro*.

As reported by Lee *et al.* (9), the canonical coiled–coil interaction motif places the core of two dimers of SFPQ approximately 100 Å apart (Supplementary Figure S1). However, a second potential interface in the crystal structure 4WIK was identified using PISA (17), involving a more distal part of the coiled–coil domain, placing each dimer core approximately 180 Å apart (Supplementary Figure S1). Analysis of this second longer interface with SOCKET (18) did not satisfy a strict definition of a coiled-coil, however, using a generous contact distance of 8.5 Å the interface was identified with marginal confidence as a coiled-coil (9). Cell-based studies by Lee *et al.* (9) demonstrated that this distal coiled–coil interaction motif of SFPQ (566–598) is important for protein localisation, presumably through oligomerisation. This region has also been identified as a ‘charged single alpha helix’ (19) in a theoretical study by Dobson *et al.* (20), where it is described as ‘highly versatile and can mediate a number of different interactions and multimerisation modes in the different proteins in a context-dependent manner’.

In this work, we measure the effects of the various coiled–coil interaction modes and extents using a tractable *in vitro* system based on truncated NONO and SFPQ variants. We capitalise on the fact that SFPQ/NONO heterodimers form preferentially in solution, and that by furnishing only SFPQ with a coiled–coil interaction motif, the predominant association/dissociation event is the dimer: tetramer equilibrium. In summary, our crystallographic and solution studies show that SFPQ/NONO heterodimers experience flexibility in the coiled–coil domain, and thus have potential for different interaction modes at the coiled–coil interaction motif. Small-angle scattering data for the QM (L535A/L539A/L546A/M549A) and R542C constructs serve as useful proxies for the extreme conditions of either ∼100% dimer or ∼100% canonical tetramer, respectively, while also explaining the potential impact of cysteine mutants observed in cancer patients and of the well-described nonA^diss^ allele. This work informs the ability to predict the disease impact of DBHS coiled coil mutants, and potentially mutations in the coiled–coil domains of other important human proteins. Our data support the observation that a wild-type protein truncated to only include the canonical coiled–coil interface forms moderate levels of simple tetramers in solution, but that the variant that is extended to include the additional interface is capable of a greater level of tetramerisation and the formation of additional larger complexes. Following these observations, the flexibility and interface plasticity of the coiled–coil domain of DBHS proteins may allow wider target selection, tolerance of flexibility in target nucleic acids, and thus a structural adaptation to their roles as critical multifunctional proteins essential to mammalian life.

## Materials and methods

### Plasmids for protein expression and purification and oligonucleotides

The construction of pCDF-11(SFPQ214-598), pCDF-11(SFPQ214-598_R542C), pCDF-11(SFPQ276-598_R542C), pCDF-11(SFPQ276-565), pCDF-11(SFPQ276-598_QM) and pETDuet-1-(NONO53-312) was described previously (21,9). Mutations in the plasmids pCDF-11(SFPQ214-598_R542C), pCDF-11(SFPQ276-598_R542C), and pCDF-11(SFPQ276-598_QM) were generated using the QuickChange mutagenesis protocol (22).

### Protein expression and purification

*Escherichia coli* Rosetta 2 (DE3) cells were co-transformed with either pCDF-11(SFPQ214-598), pCDF-11(SFPQ214-598_R542C), pCDF-11(SFPQ276-598_R542C), pCDF-11(SFPQ276-565), or pCDF-11(SFPQ276-598_QM) and pETDuet-1-(NONO53-312) to produce SFPQ/NONO heterodimers. Overnight culture growth, expression and purification steps were followed as described (9). Protein purity and disulfide bond formation were assessed with sodium dodecyl sulfate polyacrylamide gel electrophoresis carried out under reducing or non-reducing conditions (± β-mercaptoethanol).

### Sequence analysis and structure prediction

A sequence alignment of the three human DBHS paralogs and NONO-1 from *Caenorhabditis elegans* was done using full length protein sequences using UniProt. This alignment was then plotted and coloured in Jalview (23) along with the structural features of NONO-1 and SFPQ as predicted by ColabFold (24).

### Crystallisation and X-ray diffraction data collection

Crystals of the heterodimer of SFPQ (residues 214–598)/NONO (residues 53–312) were grown by sitting-drop vapor diffusion method at 20°C. SFPQ/NONO heterodimer (4 μL @ 7.8 mg/mL) was mixed with 2 μL of reservoir solution (0.1 M Tris-HCl, pH 8.5, 0.2 M ammonium sulfate, 19% PEG 3350) and equilibrated against 0.5 mL of the reservoir solution. Crystals were harvested and dipped in reservoir solution supplemented with 15% ethylene glycol before cryocooling. Diffraction data were recorded on beamline MX2 at the Australian Synchrotron (25) at a wavelength of 0.954 Å (13 keV) at 100 K. The data were processed with MOSFLM (26) and merged and scaled with AIMLESS (27) from the CCP4 suite (CCP4, 1994). Crystals belong to space group *P*2_1_2_1_2_1_ with unit cell parameters of *a* = 66.6 Å, *b* = 112.9 Å, *c* = 204.6 Å. Data collection and merging statistics are summarised in Table 1.

**Table 1.** Diffraction data and refinement statistics^a^

Data collection	
Space group	*P*2_1_2_1_2_1_
/Unit cell parameters (Å)	66.6, 112.9, 204.6
Resolution (Å)	68.21 – 2.85 (2.98 – 2.85)
No. of observations	173, 988 (21, 523)
No. of unique reflections	36, 690 (4, 446)
Completeness (%)	99.5 (99.9)
Redundancy	4.7 (4.8)
*R*_merge_ (%)	10.0 (67.3)
*R*_pim_ (%)	5.4 (37.8)
CC_1/2_	0.996 (0.627)
Average *I*/*σ* (*I*)	10.2 (2.4)
*Refinement*	
*R* (%)	23.6 (33.3)
*R*_free_ (%)	28.3 (33.7)
No. (%) of reflections in test set	1, 827 (5.0)
No. of protein molecules per asu	4
R.m.s.d bond length (Å)	0.003
R.m.s.d bond angle (°)	1.24
Average B-factors (Å^2^)^b^	71.5
Ramachandran plot^c^	
Residues other than Gly and Pro in:	
Most favoured regions (%)	95.9
Additional allowed regions (%)	4.1
Disallowed regions (%)	0
PDB code	6WMZ

^a^Values in parentheses are for the highest-resolution shell.

^b^Calculated by BAVERAGE in CCP4 suite (69)

^c^Calculated using MolProbity (31).

### Crystal structure solution and refinement

The crystal structure was solved by molecular replacement with PHASER (28), using the structure of the SFPQ homodimer (PDB code 4WII (9)) with all non-protein atoms removed, as a search model. A solution with a log-likelihood gain of 836 and a Z score of 14.7, located two dimers in the asymmetric unit. The correct amino acid sequence of NONO was introduced by mutating from the search model (SFPQ) and the extended coiled–coil domain of SFPQ (residues 527–589) were built using COOT (29) in conjunction with iterative refinement using REFMAC (30). The final model consists of two heterodimers (SFPQ residues 291 – 589 in Chain A and NONO residues 68 –307 in Chain B; SFPQ residues 293–584 in Chain C and NONO residues 54–307 in Chain D), 39 water molecules, and one sulfate ion. The quality of the model was validated using MOLPROBITY (31). The refinement statistics are included in Table 1, and the atomic coordinates have been deposited in the Protein Data Bank as entry 6WMZ.

### Small angle X-ray scattering

#### Data reduction and analysis

Small-angle X-ray scattering (SAXS) SAXS data for all SFPQ/NONO constructs were collected on the SAXS/WAXS beamline at the Australian Synchrotron using an inline SEC-SAXS sheath-flow setup (32,33) or a ‘static-SAXS’ setup where data was collected via constant flow of the sample through a capillary in the path of the beam, without the use of a SEC column. Exposures were taken at a camera length of 2680 mm, with an X-ray wavelength of 0.10781 nm with either a Pilatus 3 2M detector, or a 2D Photon counting Dectris/Pilatus 1M pixel detector (Table 2). Data on concentration series were all collected using a buffer of 20mM Tris-Cl (pH 7.5), 250mM NaCl: SFPQ276-565/NONO53-312 (ΔCSAH) from 6.8–0.32 mg/ml; SFPQ276-598/NONO53-312 (QM) from 3 to 0.4 mg/ml; SFPQ276-598 (R542C)/NONO53-312 concentrated to 0.78 or 1.6 mg/ml. Separately, SFPQ214-598(R542C)/NONO53-312 was concentrated to 6.8 mg/ml in 20 mM Tris-Cl (pH 7.5), 250 mM NaCl, and analysed on a pre-equilibrated Superdex 200 5/150 column (GE Healthcare) with UV absorbance at 260 and 280 nm monitored alongside scattering. Data reduction was carried out using Scatter-Brain version 2.82 (software for acquiring, processing and viewing SAXS/ WAXS data at the Australian Synchrotron) and corrected for solvent scattering and sample transmission. Some data processing and analysis was performed using the ATSAS suite (34). For all SEC-SAXS data, flat, self-consistent non-protein regions of the chromatogram were averaged and taken as solvent scattering with CHROMIXS. The sample scattering was then taken as the average of frames with similar *R*_g_ values that were measured as the protein eluted. The molecular mass was estimated using the Bayesian inference method in PRIMUS. Pair-distance distribution functions P(r) were generated from the experimental data using GNOM/PRIMUS.

**Table 2.** Small-angle X-ray scattering data collection parameters

	(ΔCSAH)	(QM)	R542C	R542C-DBD
Wavelength (nm)	0.10 781	0.10 781	0.10 781	0.10 781
Camera length (mm)	2680	2680	2680	3540
*q*-range (Å^−1^)	0.01–0.38	0.01–0.36	0.01–0.38	0.01–0.38
Exposure time (s)	35 1 s frames	40 1 s frames	35 1 s frames	700 successive 1 s frames
Configuration	Static-SAXS	Static-SAXS	Static-SAXS	SEC-SAXS
Concentration (mg/ml)	0.32	0.75	0.78	6.8
Temperature (°C)	25	25	25	25
Mass from sequence (kDa)	64	68	137	149
I(0) From Guinier	0.02	0.034	0.08	0.064
*R*_g_ from Guinier (nm)	2.99	3.98	5.33	5.46
I(0) from P(r)	0.02	0.03	0.08	0.06
*R*_g_ from P(r) (nm)	3.1	4.2	5.4	5.6
*D*_max_ from P(r) (nm)	12.1	16.5	21	20.4
*R*_g_ from Crystal (nm)	3.24	4.23	5.529	5.529
Experimental molecular mass (kDa)	62.35	70.55	185.78	208

Theoretical P(r) functions for crystal structures were generated by first calculating the scattering profile for a given crystal structure using CRYSOL and then applying GNOM to each calculated profile. Guinier analysis was performed using BioXTAS RAW (35). Both Guinier analysis and respective P(r) functions were used to calculate I(0), *R*_g_ and *D*_max_. Dimensionless Kratky plots were also generated in BioXTAS RAW using the automatically predicted *R*_g_ for each construct. CRYSOL was used to compare models of SFPQ/NONO heterodimers to the QM and ΔCSAH experimental scattering data using the lowest concentration curves. All data at available concentrations were analysed in BioXTAS RAW (35) for singular value decomposition (SVD). These data also included the previously published concentration series collected on SFPQ/NONO by Lee *et al.* (9). The SVD autocorrelation values were used to estimate the number of components existing in solution for each concentration series.

#### 3D modelling

DAMMIF was employed on the lowest concentration data without any symmetry constraints to model a general approximation of the QM and ΔCSAH constructs. The atomistic models from our novel 6WMZ structure were superposed over the bead models to assess the accuracy of our reconstruction. To generate an *ab initio* approximation of the SFPQ214-598(R542C)/NONO53-312 construct our SEC-SAXS data was used in the program GASBOR (34) with 1296 amino acid residues and *P2* symmetry as inputs. The programme, CORAL (34), was also used to generate 3 models with a tetrameric structure. Two dimers and one tetramer were used as initial input structures. The dimers each matched the conformationally different dimers in the asymmetric unit of 6WMZ with their respective C- and N-termini extended to fill in the residues missing from the 6WMZ structure: a small helix was added to the N-terminus and the coiled–coil domain further extended as an α-helix. The N-terminus of NONO was also extended with a small loop to residue 54 to match the other molecule of NONO in the asymmetric unit of 6WMZ. Using both conformationally different dimers as separate inputs *P2* symmetry was applied and SFPQ and NONO were set as ‘free’ but grouped together as paired domains, in order to preserve the structurally-conserved dimer interface. To constrain the output tetrameric models into an antiparallel configuration contacts were specified between opposing R542 and R542’ residues of SFPQ at 8 Å and neighbouring M549 and L535 residues in both chains of SFPQ at 15 Å. To generate a 3rd model from a tetrameric input the entire 6WMZ tetramer (with additional extended regions to fill out the construct boundaries) was set as ‘fixed’ and assigned *P1* symmetry. All models also had the missing 65 residues on the N-terminus of each chain of SFPQ filled in as random loop regions by CORAL. Reduced χ^2^ values were used to assess the agreement of each model with the experimental data, as well as normalised error-weighted residual plots. SAXS data collected at the Australian Synchrotron and reduced with Scatter-Brain typically has a 2x standard error as the error on the scattered intensity, meaning perfect fits to the experimental data have a reduced χ^2^ value of 0.25 rather than 1 (33,36). Details of the data collection and structural parameters are summarised in Table 2, and data have been deposited in the SASBDB (37) with accession codes SASDMV7 (SEC-SAXS of Tetramer), SASDMW8 (Concentration series on Tetramer), SASDMW7 (ΔCSAH) and SASDMG8 (QM heterodimer).

#### Mass spectrometry for disulfide linkages

To confirm the presence of a disulfide linkage non-reducing SDS-PAGE was performed to acquire a band of SFPQ214-598(R542C) which ran on the gel at twice the molecular weight of an SFPQ monomer. This band was excised and subjected to an in-gel digest with trypsin for mass spectrometric analysis. Briefly, protein bands were de-stained in 50% methanol/100 mM ammonium bicarbonate (Ambic). Then, the gel pieces were dehydrated in acetonitrile (ACN), and next rehydrated in 40 mM iodoacetamide in 100 mM Ambic at room temperature for 30 min in darkness. The dehydration/rehydration steps were repeated before the gel pieces were completely dried at 50°C for 5 min. The gel pieces were rehydrated in 20 μl of a 10 ng/μl trypsin in 50 mM Ambic/10% ACN on ice for 10 min. Additional 50 mM Ambic was added to completely cover the gel pieces, which were then subjected to overnight digestion at 37°C. Peptides were extracted from the polyacrylamide gel with 50% ACN-5% formic acid and 90% ACN-5% formic acid. The supernatants were combined and dried for MS analysis.

Peptides were analysed at a flow rate of 300 nl/min over a 17 min gradient (2–35% (v/v) ACN) with a self-packed 150 mm × 75 μm Dr Maisch Reprosil-Pur 120 C18-AQ 1.9 μm column driven by an Ultimate U3000 nano pump. The Orbitrap Exploris 480 MS was operated in data-dependent acquisition mode using a full scan with m/z range 375–1200, Orbitrap resolution of 60 000, normalised target value 300%, and maximum injection time set to Auto. The intensity threshold for precursor was set to 5 × 10^4^. MS/MS spectra starting from 120 m/z were acquired in DDA mode with an isolation window of 1.6 Da and subsequently fragmented with HCD using an normalised collision energy of 28%. Orbitrap resolution was set to 15 000. The normalised AGC target was 75%, and the maximum injection time was Auto.

To analyse the sequence coverage, raw files were processed using the MaxQuant software (Version 2.4.0.0) (38) using default settings. Trypsin was used as protease and up to two allowed missed cleavages. Cysteine carbamidomethylation, oxidation of methionine and N-terminal acetylation were set up as a variable modification. A reference database by adding sequences for SFPQ214-598 and NONO53-312 to the reference *E.coli* (strain B / BL21-DE3) fasta file (UP000002032) downloaded from Uniprot. Presence of disulfide bridges was analysed with SIM-XL using default settings (39).

## Results and discussion

### A crystal structure of an SFPQ/NONO tetramer: Novel interfaces and details

**Figure 1.**
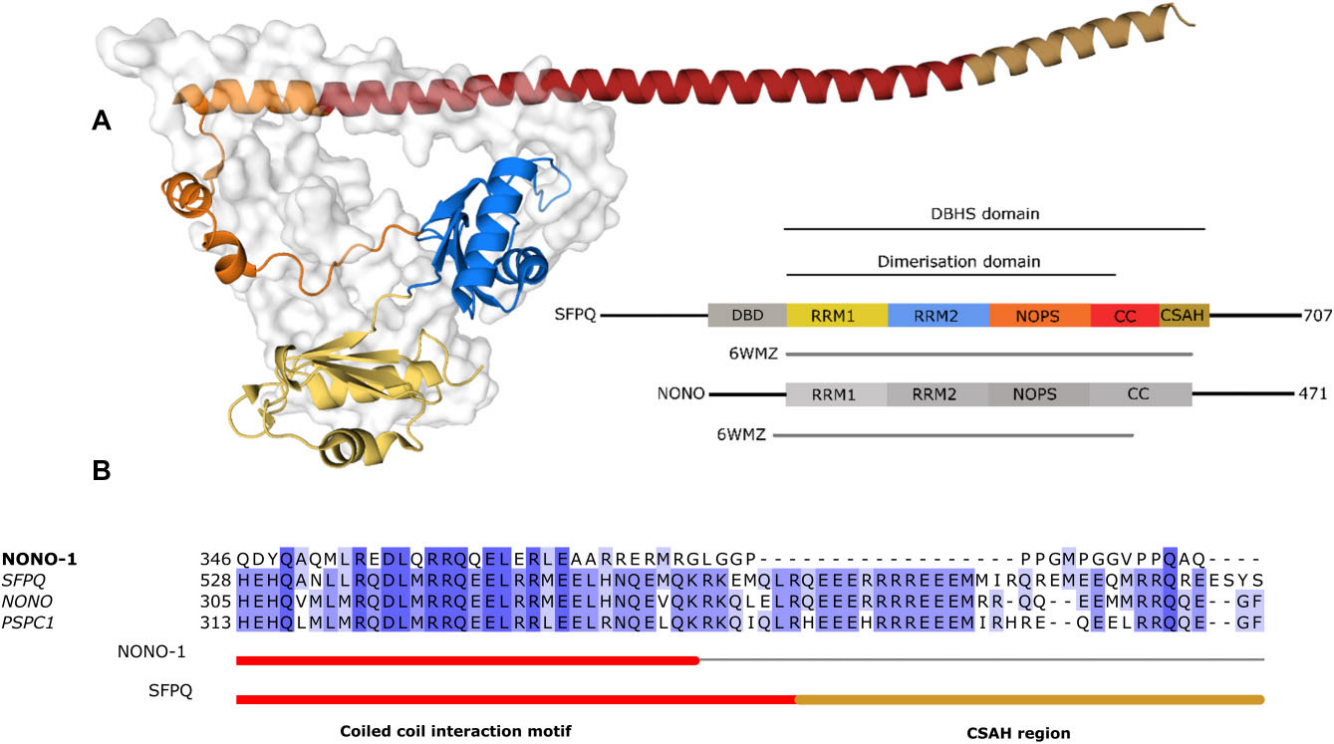
Crystal structure of an SFPQ/NONO heterodimer. (**A**) Novel crystal structure of an SFPQ/NONO heterodimer colored yellow (RRM1), dark blue (RRM2), orange (NOPS), red (coiled-coil or CC) and gold (CSAH) of SFPQ in cartoon representation. NONO is shown in surface representation. The construct boundaries used in this study are drawn and include residues 214–598 of SFPQ and 53–312 of NONO with the colours matching the crystal structure. (**B**) The aligned amino acid sequences of the coiled–coil domain in NONO-1 from C. elegans, SFPQ, PSPC1, and NONO from H. sapiens. These are colored by amino acid similarity (darker colours indicate amino acid agreement). The Alphafold predicted α-helical regions are indicated below with the canonical coiled–coil interface region in red and the CSAH region in gold. The region beyond the coiled–coil interaction motif in NONO-1 is not predicted to be alpha helical and lacks homology to the CSAH region in the human paralogs.

The overall structure of the SFPQ/NONO heterodimer is in good agreement with the previously characterised structures of the DBHS proteins ((7–11); PDB entry 7LRQ; (12); Wang *et al.*, 2022). Similar to these published structures each monomer is made up of 2 consecutive RNA-Recognition Motifs (RRM1, RRM2), a paraspeckle targeting domain (NOPS), and a coiled coil domain (Figure 1A). Likewise, the SFPQ/NONO dimer is also governed by extensive intermolecular interactions (primarily hydrophobic interactions) between parts of the dimerisation domain (Figure 1A). Despite the presence of the DNA-binding domain (DBD) of SFPQ in the protein, all copies of SFPQ in the crystal structure lack the electron density corresponding to the N-terminal residues (DBD, residues 214–290 in Chain A and 214–292 in Chain C) before the first RRM.

**Figure 2.**
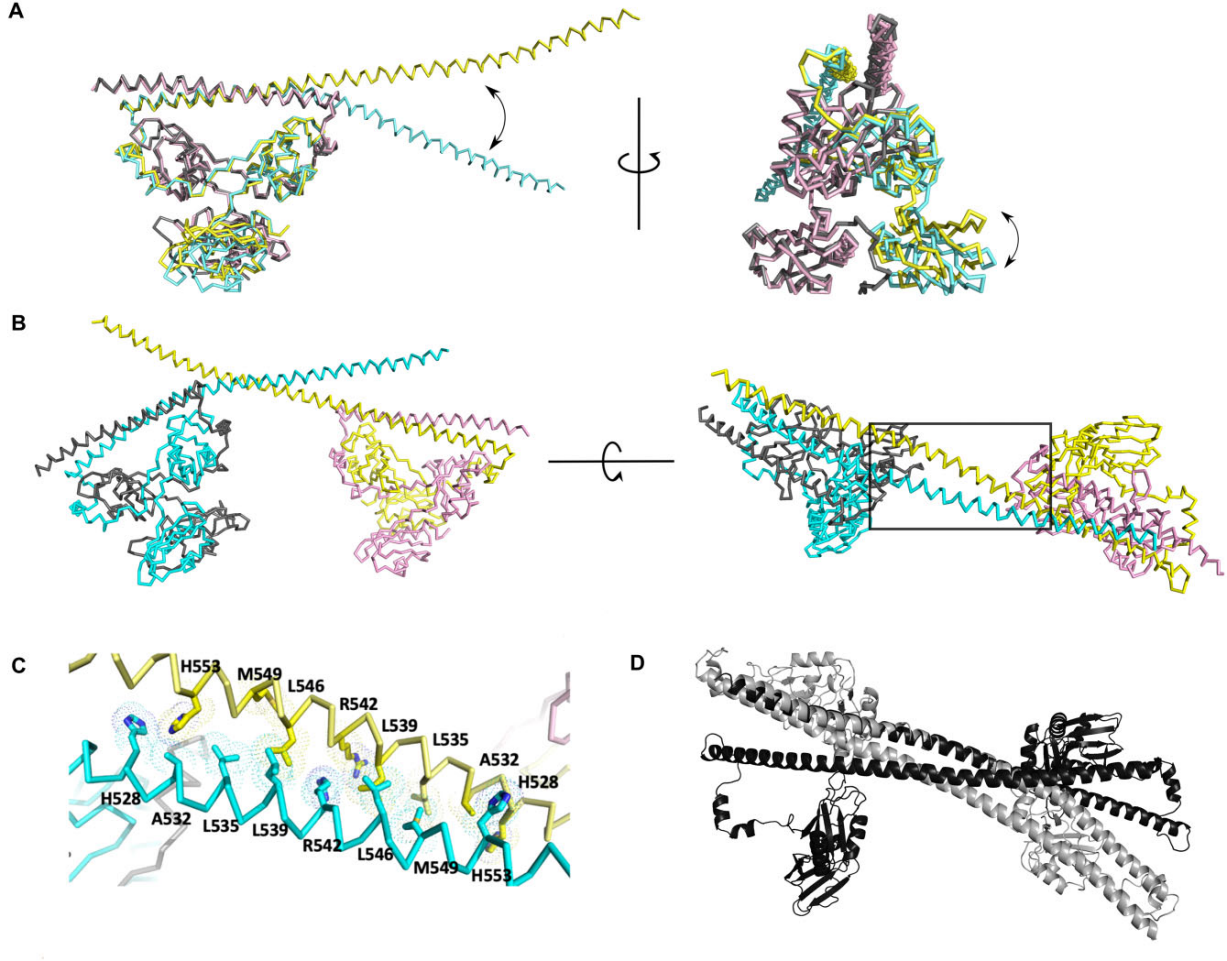
Crystal structure of an SFPQ/NONO tetramer. (**A**) Superposition of the two heterodimers of SFPQ/NONO in the asymmetric unit reveals the domain movement of RRM1 and the CC domain of SFPQ. Chain A (SFPQ) in yellow, Chain B (NONO) in pale pink, Chain C (SFPQ) in cyan and Chain D (NONO) in grey. (**B**) Tetramer interface formed in the asymmetric unit of the crystal, Chain A (SFPQ) in yellow, Chain B (NONO) in pale pink, Chain C (SFPQ) in cyan, and Chain D (NONO) in grey. (**C**) Close-up view of the CC interaction mediated dimer-dimer interface. (**D**) Comparison with the CC interaction interface with that in the SFPQ polymer (4WIJ) using the same viewpoint as in the lower panel of (**B**). The coiled–coil interaction motif (residues 528–555) of the SFPQ/NONO tetramer (black) and that in Chain A of the SFPQ homodimer tetramer structure (grey) (4WIJ) are superposed. The neighboring symmetry-related Chain B (y + 1/2, -x + 1/2, z + 1/4) of the SFPQ homodimer structure (4WIJ) is shown while NONO is omitted in this figure for simplicity.

Studies have previously noted the significant degree of conservation in the region C-terminal to the coiled–coil interaction motif and made inferences about the function of this region (20,40,9). For this study, we term this region the Charged Single Alpha Helix ‘CSAH’ region (20) (Figure 1). The resulting crystal contains two copies of SFPQ/NONO heterodimers in the asymmetric unit (Figure 2A). As previously observed in the structures of PSPC1/NONO and SFPQ/PSPC1 heterodimers (7,8,10), similar pseudosymmetry between SFPQ and NONO is observed within the dimerisation domain.

However, due to the presence of the extended coiled–coil domain in SFPQ, the resulting heterodimer is asymmetrical beyond the dimerisation domain. The major disparity between the two copies of the SFPQ/NONO heterodimers in the asymmetric unit is contributed by the dispositions of RRM1 and the extended CC (Figure 2A and Supplementary Figure S2), reinforcing the previous observations on the domain movement and the plasticity of the CC domain (20,41,8,9). The relative position of RRM1 of SFPQ in Chain C to that in Chain A is tilted by 27.6° while the CC domain of SFPQ in Chain C is rotated by 35° relative to that in Chain A. The extended CC of SFPQ interacts with that of SFPQ in the second copy of the heterodimer, resulting in the formation of an SFPQ/NONO tetramer (Figure 2B). This is consistent with our previous findings that the coiled–coil interaction motif (residues 528–555) in the extended coiled coil mediates the polymerisation of SFPQ, which is critical for the function of SFPQ in paraspeckle formation and transcriptional activation (9). Closer inspection of the interface generated by the antiparallel coiled–coil interaction revealed notable differences in the molecular interactions in the interface from that reported previously for the SFPQ homodimer (4WIJ, (9)) (Figure 2C and D). When the coiled–coil interaction motifs (residues 528–555) of Chain A from the SFPQ homodimer (4WIJ) and SFPQ/NONO heterodimer (6WMZ) are superposed, the disposition of the partnering coiled-coil motif in the interface is significantly different, rotated by 38.6° and displaced by 9.6 Å (Supplementary Figure S3 and Supplementary Figure S4). The angles between the coiled–coil interaction motifs in the SFPQ/NONO tetramer and in the SFPQ homodimer polymer are 150.3° and 157.0°, respectively. This displacement results in a reduced interface area of 844 Å^2^ in the SFPQ/NONO tetramer structure in comparison to that in the homodimeric polymer interface area being 1089 Å^2^. Despite the reduction in the interface area, the solvation energy gain (Δ^i^G) for this interface in the SFPQ/NONO heterodimer structure is increased to −9.7 kcal mol^−1^ (*P* = 0.214) from −7.4 kcal mol^−1^ (*P* = 0.329) in the homodimeric polymer interface, indicating stronger hydrophobic interaction. The difference in the CC interaction is highlighted by the C*α* position of R542 in the centre of the coiled–coil interaction motif (Supplementary Figure S3). The C*α*-C*α* distances of the hydrophobic residues in the coiled–coil interaction motifs are noticeably different (Supplementary Figure S3 and Supplementary Table S1). For example, L539 in Chain A shares the shortest C*α*-C*α* distance (7.6 Å) with L539 in the symmetry-related Chain B (y + 1/2, -x + 1/2, z + 1/4) in the SFPQ homodimer structure while L539 in Chain A in the SFPQ/NONO heterodimer has the shortest C*α*-C*α* distance (7.2 Å) with L546 in Chain C.

### SFPQ coiled–coil domain in solution: Scattering data and flexibility

Given the limitations of crystallography for studying conformationally flexible proteins and to investigate the prevalence of various coiled-coil configurations in solution, small-angle X-ray scattering in the form of both concentration series and SEC-SAXS experiments was performed on the SFPQ mutants, R542C, QM (L535A/L539A/L546A/M549A) and the ΔCSAH variant in the context of SFPQ/NONO heterodimers. All data collection parameters and a comparison of key experimental features to those derived from our crystal structure are presented in Table 2:

Given the reported dimer-tetramer equilibrium via the coiled–coil interaction motif (9), we hypothesised that the R542C mutant should act as a constitutive tetramer, and the QM mutant as a constitutive dimer, representing the extremes of the equilibrium.

**Figure 3.**
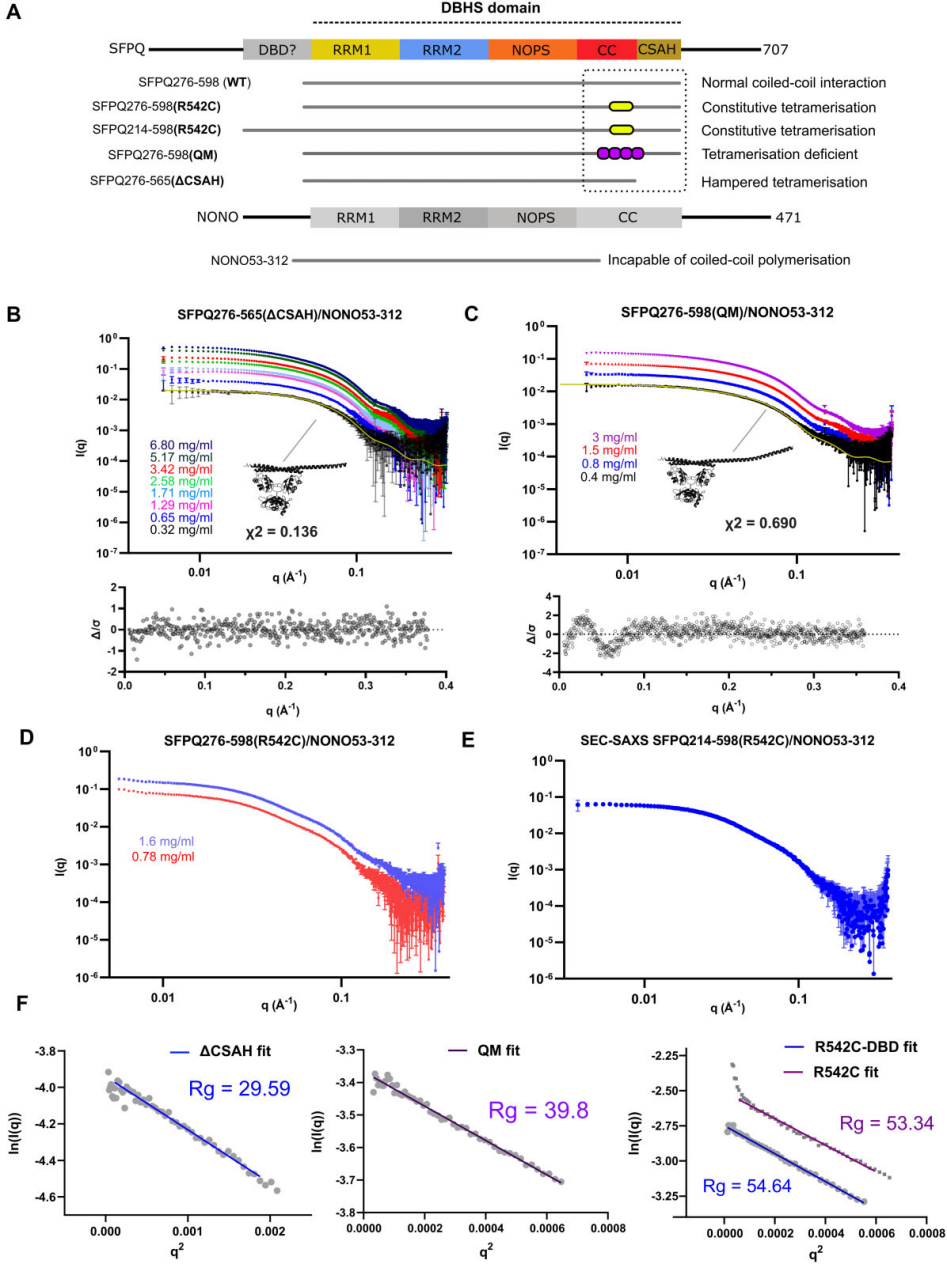
Solution studies on protein variants of SFPQ/NONO (**A**) Boundaries of all the SFPQ constructs used in SAXS experiments and attached descriptions of their observed behaviour in solution. (**B**) Log(**I**) versus Log(q) plot for SFPQ276-565/NONO53-312 (ΔCSAH) alongside a residual plot describing the CRYSOL fit of a truncated 6WMZ model to the data. (**C**) Log(**I**) versus Log(q) plot for SFPQ276-598/NONO53-312(QM) alongside a residual plot describing the CRYSOL fit of a 6WMZ model to the data. Systematic wavelike deviations in the residuals plot are likely caused by movement of the coiled–coil domain in solution (**D**) Log(**I**) versus Log(q) plot for SFPQ276-598(R542C)/NONO260. (**E**) Log (**I**) versus Log(q) plot for SFPQ214-598(R542C)/NONO53-312. Data were captured using static-SAXS and SEC-SAXS, respectively. (**F**) Guinier fits calculated in BioXTAS RAW with the associated R_g_ values for each construct in angstroms. The ΔCSAH residuals were flat across q.R_g_ 0.3119–1.2822 suggesting a globular particule. The QM residuals began to uptick at q.R_g_ > 1 and so the Guinier fitting range was adjusted to q.R_g_ 0.228–1.01 and was linear suggesting a partly rodlike particle. The SEC-SAXS data (R542C-DBD) showed flat residuals over the q.R_g_ range 0.201–1.284, suggesting a globular particle. Given this q.R_g_ max was set as 1.3 for the R542C alone fit and the first five data points excluded from the fit (35). However, the residuals still showed a characteristic smile pattern suggesting aggregation.

This notion was supported by the presence of bands of SFPQ that ran at twice the predicted MW of a SFPQ monomer in the absence of reducing agent (Supplementary Figure S5). The addition of a reducing agent caused these bands to run at the MW of a monomer (Supplementary Figure S5). Further mass spectrometry analysis also indicated the presence of a disulfide bond between SFPQ C542 containing peptides and not the reactive solvent exposed cysteine in NONO C145 (42) (Supplementary Figure S8). Log(*I*) versus log(*q*) plots for all four experiments (Figure 3B–E) indicate good quality scattering data.

**Figure 4.**
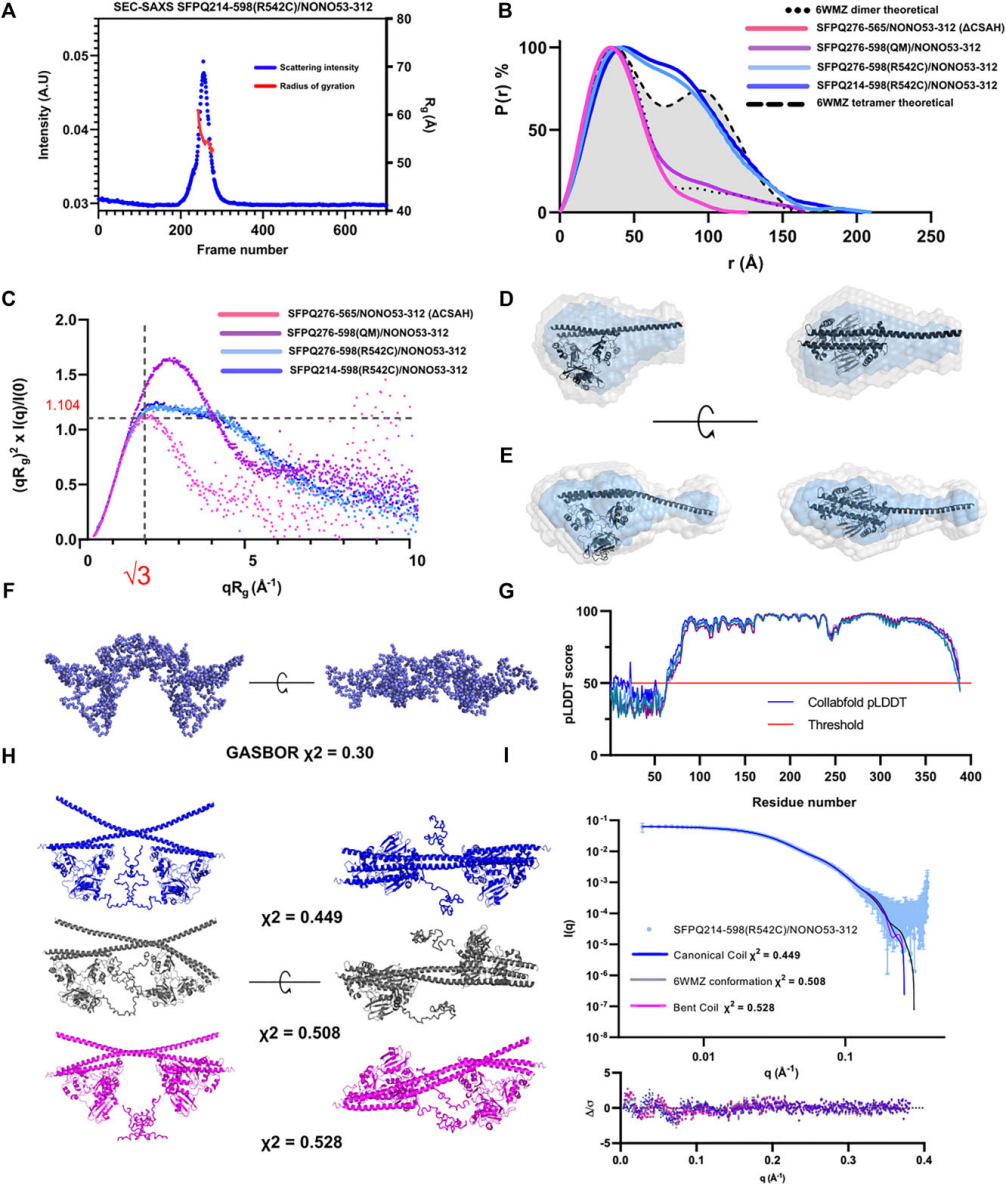
Dimers and a constitutive tetramer of SFPQ/NONO (**A**) CHROMIXS SEC-SAXS chromatography trace for SFPQ214-598(R542C)/NONO53-312. Scattering intensity is plotted in blue with the estimated radius of gyration plotted in red. (**B**) P(r) functions for the constructs considered amenable to 3D reconstruction plotted against theoretical distributions to assess homogeneity and structure. (**C**) Kratky plot of 3D modelling candidates to assess foldedness and globularity. (**D**/**E**) DAMAVER (grey) and DAMFILT (cyan) models of the QM and ΔCSAH constructs. Black crystal structure of the 6WMZ dimer with a full-length coiled–coil domain and truncated version superposed over the bead models. (**F**) GASBOR model generated from the SFPQ214-598(R542C)/NONO53-312 dataset demonstrates that the data roughly correspond to the shape of a tetramer. (**G**) pLDDT scores for different models of SFPQ214-598 produced in CollabFold. This can be considered a relatively strong indicator that the first 65 residues of this construct are likely disordered (scores below or close to 0.5). (**H**/**I**) CORAL fits to the data of SFPQ214-598(R542C)/NONO53-312 demonstrate that a variety of input structures demonstrate different fits to the data. The blue model is derived from the dimer in the asymmetric unit of 6WMZ, which has a relatively straight coiled–coil domain. The grey model is the 6WMZ tetramer fixed as a rigid body. The pink model uses the other dimer from the asymmetric unit of 6WMZ which has a bent coiled–coil domain. Modelling constraints reflected a disulphide bond between coiled–coil domains as well as adjacent residues kept at ∼15 angstroms to mimic a canonical antiparallel coiled–coil interaction. Normalised residual plot indicating systematic deviations of the fit from the data, most likely caused by domain flexibility.

All constructs apart from the static-SAXS experiment on the R542C mutant appeared to have a linear Guinier region with a predictable relationship between scattering intensity and protein concentration (linear fit up to *q.R_g_*max ∼ 1.3*)*. The R542C mutant static-SAXS data displayed a slightly curved Guinier residual plot perhaps due to some aggregation. Guinier analysis indicated that the QM data were likely a partly rodlike particle *(*Linear fit up to *q.R_g_* max of ∼ 1*)* whilst the ΔCSAH and R542C constructs appeared globular. We report that a model of an SFPQ/NONO dimer derived from our crystal structure, with the CSAH region truncated, has an acceptable fit to the data, with some small amount of overfitting as indicated by a reduced χ^2^ of 0.136 in place of 0.25 (Figure 3B). The general features of the experimental data conform to the expected parameters of a ΔCSAH SFPQ/NONO dimer (Table 2, Figure 4B). The fit of untruncated dimer against the QM data revealed a more elevated reduced χ2 value with systematic deviations at low/mid-*q* in the error-weighted residual difference plot (Figure 3C). Given low-to mid-*q* regions in SAXS data typically contain information about sample shape, domain positioning, and orientation (36) these deviations can be taken as an indicator of the flexibility of the coiled–coil domain in solution. However, they may also be the result of additional species in solution, such as a small amount of aggregate or the presence of tetramers.

### SFPQ coiled-coil behaviour in solution: Dimers and constitutive tetramers

The SEC-SAXS data for SFPQ214-598(R542C)/NONO53-312 showed the formation of a single peak in the chromatogram with a shoulder on its leading edge (Figure 4A).

The centre and trailing edge of the peak have a stable *R*_g_ of 55 Å. UV spectrophotometry confirmed the peak had a 260:280 nm absorbance ratio consistent with that of a protein solution (Supplementary Figure S7). Pairwise distance distribution plots were calculated to evaluate the effect of the QM and R542C mutations on protein structure and assess the potential for 3D reconstruction (Figure 4B). The distance distribution for the QM construct at a concentration of 0.38 mg/ml closely mimics the theoretical distribution of an SFPQ/NONO dimer (Figure 4B).

The distribution for SFPQ276-598(R542C)/NONO53-312 at both concentrations collected via batch-SAXS closely mirrors that of SFPQ214-598(R542C)/NONO53-312 collected via SEC-SAXS. The distributions for both these proteins appear to be bimodal and much larger than the experimental and theoretical distributions of a dimer (Figure 4B). Additionally, they match the size and shape of the theoretical distribution of a tetramer calculated using CRYSOL (Figure 4B). Calculated *R*_g_, *D*_max_, and MW values from crystal structures and solution data are also commensurate with the QM data corresponding to dimer, and the R542C data to tetramer (Table 2). Three of our constructs appear folded and mostly globular in the dimensionless Kratky plot (Figure 4C). However, the QM data at 0.32mg/ml does not peak at y = 1.104, x = √3. This is likely due to the sample being a mixture of a rod-like and globular particle and the coiled–coil domain flexing in solution. The dimensionless Kratky plot for the ΔCSAH construct at 0.32 mg/ml peaks at y = 1.104, x = √3 reinforcing the notion that the disparity is caused by contributions to scattering from the extended coiled–coil domain. The datasets for the QM, ΔCSAH dimers, and the R542C tetramer were deemed to be monodisperse and conformed to the general criteria required for *ab initio* 3D reconstructions (36). DAMMIF produced several models which conformed to the expected shape of an SFPQ/NONO dimer (Figures 4D, E). Logically, the dimensions for the ΔCSAH dimer are not as elongated as in the QM model (Figures 4B,d and E). Our *ab initio* reconstruction using GASBOR from the SFPQ214-598(R542C)/NONO53-312 data revealed a model which conforms to the expected shape of a tetramer (Figure 4F). The model had a good fit to the data with a reduced χ^2^ value of 0.30. However, given our host of available crystal structures of SFPQ and NONO three atomistic models were generated by CORAL. The model which conforms to the canonical 4WIJ interface the most (Figure 4H) has the least discrepancy with our data. A model using the 6WMZ tetrameric interface (Figure 4H) and a novel interface theorised by using two dimers with bent coiled–coil domains from the 6WMZ structure have greater discrepancies with the data. The imperfect fit to the data which can be seen in the systematic deviations in the normalised residuals plot (Figure 4I) are likely caused by rotational flexibility about the disulfide bond and flexibility of the unstructured DBD. It is likely given the radically different positions of the coiled coil domain we have simultaneously observed in our crystal structure across dimers in the asymmetric unit that this protein variant behaves in solution as a mixture of such tetrameric models. Each model is consistent with the R524C mutation triggering the constitutive tetramerisation of SFPQ via the formation of a disulfide bond between opposing cysteines in the coiled–coil domain.

### SFPQ coiled-coil behaviour in solution: Oligomerisation and mutation

**Figure 5.**
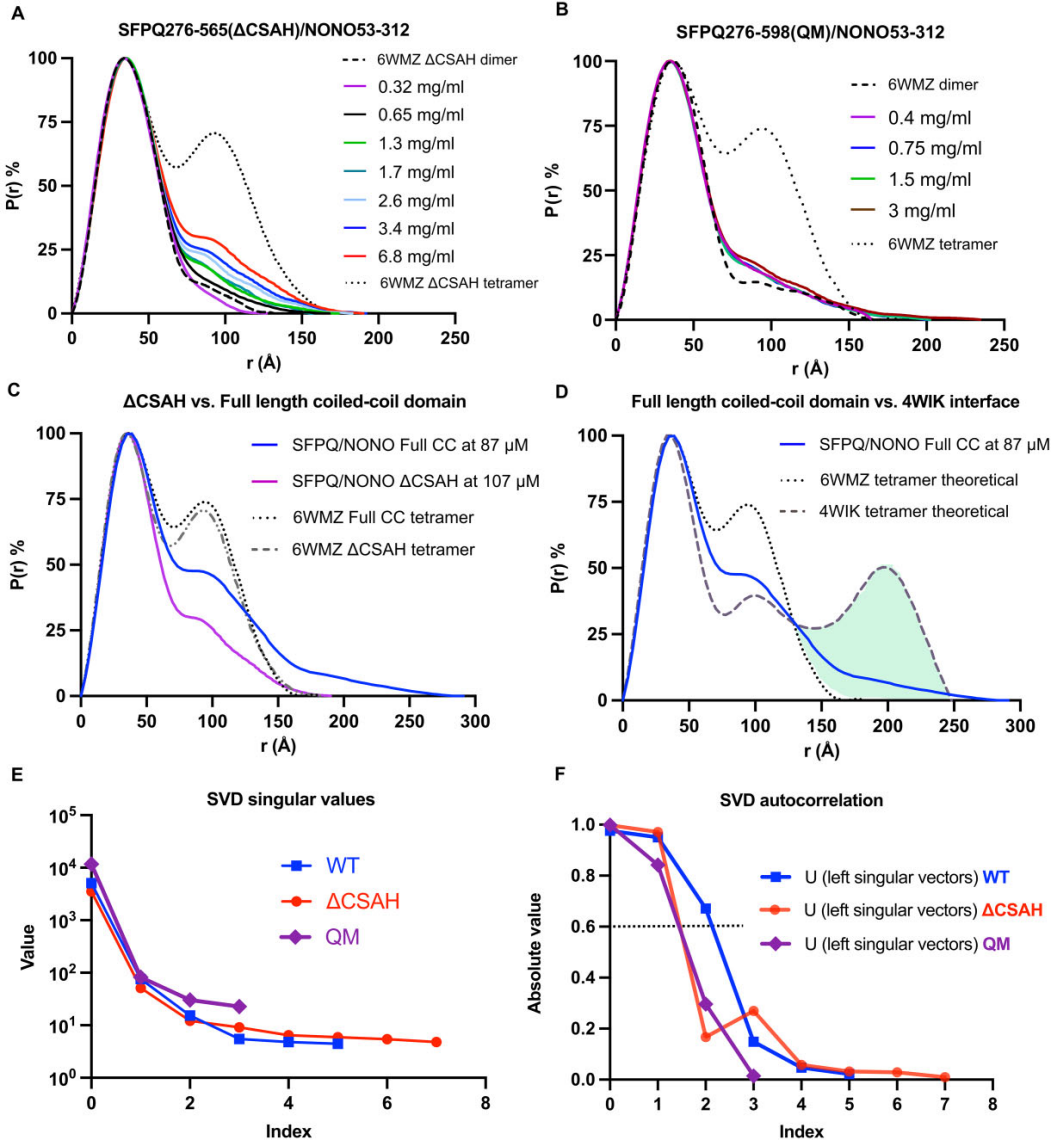
Distance distribution analysis of SFPQ variants and SVD (**A**) P(r) distribution of the SFPQ276-565/NONO53-312 dataset, curves indicate a change in structure as a function of concentration that resembles tetramerisation (9). Theoretical distributions are plotted alongside the data as a reference. (**B**) P(r) distributions for the SFPQ276-598/NONO53-312 (QM) dataset. Data indicate some concentration-dependent changes. (**C**) P(r) distributions for SFPQ276-565/NONO53-312 (ΔCSAH) and SFPQ276-598/NONO53-312 (WT) at comparable concentrations, a smaller distribution for SFPQ276-565/NONO53-312 at a higher concentration corresponds to a reduction in tetramerisation. (**D**) P(r) distribution for a dataset of SFPQ276-598/NONO53-312 plotted against the theoretical distributions of the 6WMZ tetramer and a tetramer formed via the non-canonical 4WIK interface. Green highlights represent predicted distances present in the experimental data and the 4WIK interface but absent from the theoretical distribution of a canonical tetramer. (**E**) Singular values obtained by SVD in BioXTAS Raw for all scattering experiments. (**F**) SVD autocorrelation results from BioXTAS RAW. Every data point above an absolute value of ∼0.6 is an estimated unique scattering species in solution.

To examine the relevance of our coiled-coil variants to the oligomerisation of SFPQ the *P*(*r*) functions at different concentrations were compared to theoretical functions calculated using CRYSOL/GNOM. Evidently, the ΔCSAH variant is still capable of forming tetramers as seen by the rise in distances at 100 Å relative to the theoretical distribution (Figure 5A). This is to be expected as the protein retains the essential coiled–coil interaction motif (9). The data for the QM concentration series appears to display a weak concentration-dependent interaction, however, remains largely dimeric over the examined range (Figure 5B).

Scattering from wild-type and ΔCSAH constructs was compared to assess the contribution of the CSAH region to concentration-dependent polymerisation (Figure 5C). Intriguingly, the distance distribution plot for the ΔCSAH construct at a higher concentration has a significantly smaller area than that observed for the wild-type protein. Tetramers formed by the wild-type or ΔCSAH SFPQ/NONO variants share a similarly sized theoretical distribution (Figure 5C). This demonstrates that the ΔCSAH construct has a reduced ability to form tetramers. Theoretical distance distributions calculated from atomic models and experimental distributions for the R542C variants demonstrate that the maximum dimension (*D*_max_) of a tetramer binding via the coiled–coil interaction motif should be in the order of ∼200 Å (Figure 5B). However, multiple scattering replicates (9) of the wild-type protein at concentrations upwards of 5mg/ml have produced *D*_max_ values of greater than or equal to 250 Å (Figure 5D). Simulating a P(r) function for an SFPQ/NONO tetramer binding via the CSAH interface using CRYSOL reveals an overlap with the longer distances observed in our experimental data (Figure 5D).

We suspect this is because the extended part of the coiled coil domain is facilitating the formation in solution of a larger extended interface as observed in the SFPQ369-598 crystal structure 4WIK (9) alongside the canonical interface. Given this information, we posited that perhaps the historical data for the wild-type protein which was initially identified as a two-state system (9) was potentially a three-state system. Analysis of the wild-type dataset using SVD estimated that perhaps three species are present in solution (Figure 5E, F). Conversely, the QM and ΔCSAH datasets are estimated to only contain two unique species in solution. Based on these observations it is evident that loss of function mutations or truncations to the coiled–coil domain of SFPQ reduce the predicted number of species in solution. This suggests that the wild-type protein may be capable of forming longer, polyvalent structures in solution which could result in networks or fibres (9).

Analysis of the extended CSAH interface in the crystal structure of SFPQ369-598 demonstrates that the interaction occurs between elements from the CSAH region (SFPQ566-598) with the opposing coiled–coil interaction motif (Supplementary Figure S1). Truncating the CSAH region results in a total loss of contact between molecules of SFPQ in this extended crystallographic interface but not the canonical interface (Supplementary Figure S1). Interestingly, mutated residues in the QM variant should not directly affect the CSAH-mediated interface, which may explain the presence of two predicted species in the QM dataset as opposed to only one predicted by the disruption of the canonical coiled-coil.

## Discussion

### Coiled–coil domain flexibility and relevance to nucleic acid binding

Polyvalent dynamic interactions are an important biophysical characteristic of the DBHS protein family, as is often the case with proteins involved in nuclear organisation, and choreographing the post-transcriptional control of gene regulation, through biomolecular condensation (43,44). These kinds of interactions are also challenging to study using structural biology methodology. Previous crystallographic studies have revealed some of the interaction modes of SFPQ, but with some questions about their biological relevance (9). In this work, we infer further plasticity and flexibility in the coiled–coil domain of SFPQ from a new SFPQ/NONO heterodimer crystal structure, and sought to use SAXS measurements to analyse the relevance of this behaviour to the protein in solution.

A combined analysis of solution data and a variety of crystal structures (6WMZ; (41,8,9)) now shows that the DBHS coiled-coil can adopt a number of different conformations that most likely represent a continuum of arrangements that can be accessed through flexibility of the α-helix. This flexible region is a feature present in all the family members and is likely of wider structural importance. Prior evidence demonstrated that SFPQ polymerises across nucleic acids: laddering in EMSAs, crystal coiled-coil contacts of an ‘infinite’ polymer of SFPQ, a multitude of documented binding sites across different DNA and RNA types, and positively-stained electron microscopy of SFPQ-DNA filaments (1,9). An obvious explanation for the flexibility of the coiled–coil domain is that a flexible domain samples more conformations in solution and so through thermal motion is more likely to encounter another coiled–coil domain and interact. However, an additional possibility is that domain flexibility assists DBHS dimers to conform to the varying flexibilities of different larger nucleic acid substrates whilst simultaneously preserving critical filament-forming coiled–coil interactions.

**Figure 6.**
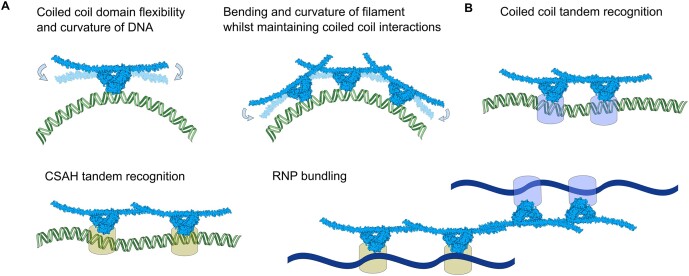
The contribution of a multiple flexible coiled–coil interfaces to nucleic acid binding (**A**) A cartoon indicating, relative flexibilities of DBHS dimers and DNA, and DBHS-DNA filaments assisted by local flexibility in the coiled–coil domain. (**B**) tandem recognition of nucleic acids sites by the canonical coiled-coil ‘molecular ruler’, tandem recognition of nucleic acids sites by the hypothesised CSAH ‘molecular ruler’ and a hybrid interaction schematic allowing bridging of DBHS-nucleic acid complexes as posited by the birds nest model of Jiang *et al.* (56).

Nucleic acids have varying persistence lengths (lp): dsDNA and dsRNA have an average lp of about ∼54 and ∼62 nm, respectively (45) whilst ssDNA and ssRNA can have lp values of 1–6 nm depending on a variety of conditions (46). DNA duplexes are also prone to bending at shorter lengths than the established persistence length of dsDNA when their sequence is made up of repeat stretches of 4–6 or more As or Ts (47–50). The ∼20-nm-long, 60-bp GAGE6 dsDNA oligonucleotide target of SFPQ ((9,51); Wang *et al.*, 2022) is a reportedly highly specific binding site for SFPQ, as only this region of the fragmented 2241-bp *GAGE6* promoter was bound by SFPQ. Previous studies have demonstrated the requirement for the coiled coil domain and the DBD in this interaction ((9); Wang *et al* 2022). The 60-bp GAGE6 oligo has AT-rich tracts so likely experiences enhanced curvature and flexibility in solution despite being significantly shorter than the standard persistence length of dsDNA. Additional shortening of the 60bp oligo by Wang *et al.* (2022) into 40, 30 and 20-mers significantly reduced the binding of SFPQ. Perhaps because 60bp is the optimal DNA length for two dimers of SFPQ to fit across the duplex and cooperatively contribute to the avidity of the interaction via flexible interacting coiled coil domains that can accommodate for the bending and flexibility of such a substrate. It seems likely that in order to form protein filaments, for a host of substrates the flexibility of the DBHS coiled–coil domain is required to contribute to the net curvature of these structures whilst maintaining points of contact with the nucleic acid (20) (Figure 6A). Interestingly, many transcription factors can recognise kinks in DNA (52) for reasons related to base/groove access and transcriptional regulation, which could be the case for SFPQ–DNA complexes.

While little is known about the detailed structure of the long non-coding RNA NEAT1_2, as it has both its 5′ and 3′ termini localised to the ‘shell’ of paraspeckles (53), there is likely to be a substantial degree of curvature in the ‘core’ region. Yamazaki *et al.* (54) have shown that NEAT1_2 has functional subdomains throughout the middle of the lncRNA which bind SFPQ and NONO intensively and are critical for paraspeckle formation. Removal of these subdomains in NEAT1_2 impeded the formation of ordered paraspeckles which could then be rescued by their incremental re-addition or the artificial tethering of DBHS proteins to the RNA. This demonstrates that the binding of DBHS proteins to NEAT1 is critical for paraspeckle formation (54,55). It is evident from CLIP data and gels that both SFPQ and NONO appear to bind across adjacent stretches of NEAT1_2, potentially through a coiled-coil polymersation mechanism (54,56,57). This may be another example of the flexibility of the coiled–coil domain compensating for or encouraging the flexing and bending of nucleic acids.

### Canonical, constitutive and non-canonical coiled–coil interfaces

In addition to the conventional coiled-coils involved in DBHS protein dimerisation and oligomerisation, the additional CSAH interface may serve to further facilitate nucleic acid recognition by enhancing the capacity of DBHS proteins to form polymerised networks. One possibility is that the CSAH region serves to allow dimer-dimer contacts to occur at a greater distance causing an initial transient CSAH-mediated approach of molecules which can stabilise into the tighter, closer, canonical coiled–coil interface. Another possibility is that the CSAH interface serves as an additional ‘molecular ruler’ for the recognition of tandem nucleic acid target sequences. The transcription factor CueR has a dimeric coiled–coil interface that allows monomers of the protein to sit at a length of 55 Å from each other (58) to keep the proteins dimerised whilst simultaneously binding to successive major grooves of DNA, and so DBHS proteins may be able to undertake tandem recognition of nucleic acids with diverse spacings via CSAH-mediated oligomerisation and canonical coiled-coil oligomerisation (Figure 6B). Interestingly, studies have documented that the methylation of arginine residues in the CSAH region of NONO reduces the protein's ability to bind dsRNA (59). Mapping these methylated residues onto SFPQ shows they are not directly involved in the longer CSAH-mediated crystal interface despite being part of the CSAH region. This raises the possibility that even more transient interfaces exist as predicted by Dobson *et al.* (20). It is a possibility that the CSAH interaction is relatively transient in the context of our experiments and that additional components are required to stabilise it *in vivo*. In the case of crystal structures containing the entire DBHS domain the crystallisation process has ‘trapped’ only the more contextually stable canonical interaction. The incremental rotation of dimers to form a larger polymer as predicted by Dobson *et al.* (20) in a superhelical model or as seen in the multiple different crystal lattices of Lee *et al.* (9) may also be relevant to nucleic acid binding: Given there are multiple types of RNA often found in paraspeckles and that each SFPQ dimer possesses four RRMs and two DBDs large multidirectional polymers where binding domains are facing outwards may help stabilise bundle-like RNP structures. This would allow SFPQ/NONO to bridge parts of the same RNA molecule or simultaneously interact with multiple different RNA molecules (Figure 6B) ((56); Wang *et al.*, 2022). In the broader context of the biologically-important liquid-liquid phase separation behaviour of SFPQ (3), the availability of a variety of interaction interfaces with proteins or nucleic acids, with different affinities, dynamics and specificity is likely important.

We show that the R542C mutation can drive the constitutive tetramerisation of SFPQ/NONO heterodimers via the canonical coiled-coil region, and given that SFPQ can form extended polymers (9) such a mutation could result in large-scale constitutive polymerisation in oxidising environments. Given the observation of zinc-driven constitutive polymerisation of SFPQ (4) in neurons leading to the imbalanced nucleocytoplasmic distribution of SFPQ, constitutive polymerisation of the cysteine variant of the *nonA^diss^* allele in flies makes for a rational explanation for the phenotype described by Rendahl *et al.* (16). This common structural rationale may also be relevant to other cysteine mutations observed in the coiled–coil domains of DBHS proteins.

Querying the databases ClinVar (60), COSMIC (61), GnomAD v4 (62) and AllOfUs (63) revealed a multitude of cysteine mutations in the coiled–coil domains of SFPQ, NONO and PSPC1 (these are summarised in Supplementary Table S2). The COSMIC database contains records of an R319C mutation in NONO which has been isolated from 3 separate tumour samples (64,65). Additionally, ClinVar lists an R327C mutation of unknown significance in PSPC1 associated with inborn genetic diseases. Based on sequence alignments these mutations are the equivalent of the R542C mutation in SFPQ (Figure 1B, Supplementary Figure S6). Given NONO’s involvement in the progression of certain cancers (66) disulfide-mediated constitutive oligomerisation of NONO may provide a molecular explanation for those specific tumour samples. Interestingly, these databases also contain records of additional disease-associated cysteine mutations in both the coiled–coil interaction motif and the CSAH region of all the DBHS paralogs (Supplementary Figure S6, Supplementary Table S2). Given the flexible and dynamic nature of DBHS coiled-coils these mutations may also be capable of inducing constitutive polymerisation, providing a molecular basis for their associated pathology. The Genome Aggregation Database (gnomAD) (62) contains some rare cysteine mutants in the coiled–coil domains of DBHS proteins which to date have not been associated with any pathology but through cross examination with other disease associated variants and the different DBHS paralogs may still be relevant to various disease states (Supplementary Figure S6, Supplementary Table S2). GnomAD v2.1.1 indicates the DBHS proteins are depleted for loss of function and missense mutations, meaning the 3 proteins are both loss of function and missense constrained with higher regional missense constraint (67) in the coiled–coil domains of NONO and PSPC1 (data not available for SFPQ), suggesting an intolerance of these proteins and regions to variation.

The NONO mutation G391C has been shown to be involved in intellectual disability and cardiomyopathy (68). Structurally, this places a cysteine residue in the beginning of a low complexity region immediately C-terminal to the coiled–coil domain. It may also be possible that a cysteine in the context of flexible and solvent exposed regions, which are adjacent to the coiled coil domain may also trigger the formation of some disulfide bonds either with other molecules of NONO via the opposing G391C, the solvent exposed residue C145, or available cysteines in other important cellular proteins, thus hindering the function of NONO. This could be relevant to other observed cysteine mutations in the C-terminal low complexity domain of DBHS proteins (Supplementary Table S2).

In summary, we have experimentally observed variety and flexibility in the coiled–coil domain of SFPQ which likely serves to facilitate and then preserve cooperative coiled–coil interactions whilst also contributing to the potential curvature, kinking and flexibility of DBHS nucleoprotein filaments. Our data revealed the formation of longer species in solution, mediated by the distal CSAH region of the coiled–coil domain, highlighting the potential relevance of a previously identified interface to cooperative nucleic acid binding. We have also demonstrated that the R542C mutation in SFPQ triggers constitutive polymerisation, serving as a useful proxy for a fixed SFPQ/NONO tetramer in solution whilst also providing a rationale for the phenotype of the *nonA^diss^* allele in *Drosophila* and perhaps other related pathologies. It is likely the observed flexibility and interface plasticity in the coiled–coil domain is important for DBHS oligomerisation, nucleic acid binding and assembly of paraspeckles within the eukaryotic cell nucleus.

## Supplementary Material

gkae1198_Supplemental_Files

## Data Availability

ΔCSAH: https://www.sasbdb.org/data/SASDMW7/ R542C: https://www.sasbdb.org/data/SASDMV7/ R542C: https://www.sasbdb.org/data/SASDMW8/ QM: https://www.sasbdb.org/data/SASDMG8/

## Crystal structure data availability (PDB)

SFPQ/NONO: https://www.rcsb.org/structure/6WMZ
