# Supplementary material for: Structural plasticity of the coiled–coil interactions in human SFPQ
Source: Nucleic Acids Res. 2024 Dec 19;53(2):gkae1198. doi: 10.1093/nar/gkae1198 (PMC11754644; doi:10.1093/nar/gkae1198)
Supplement: gkae1198_Supplemental_Files [file gkae1198_supplemental_files.zip › Suppinfo.pdf]

Supplementary figures:

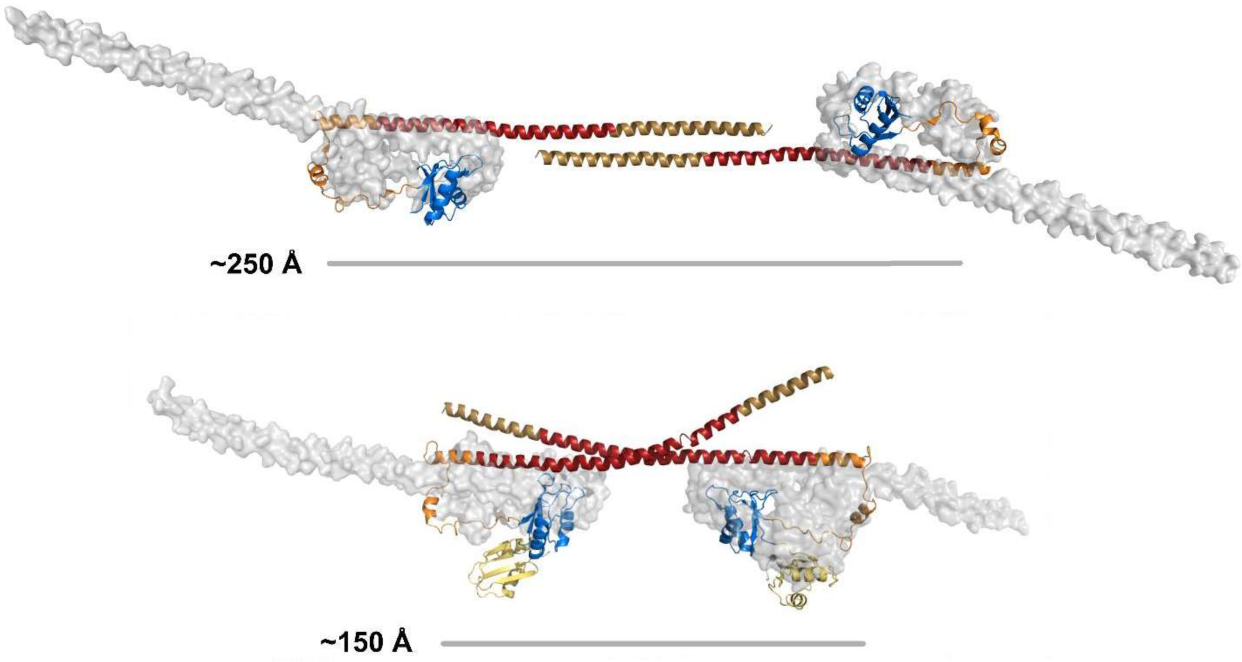

Supplementary Figure 1: **The two interfaces formed by the coiled-coil domain in crystal structures 4WIK (top) and 4WIJ (bottom).** *The resulting interfaces use different parts of the coiled-coil domain to interact and cause the core region of each dimer to sit at different lengths from one another. The CSAH region is coloured in gold in both structures.*

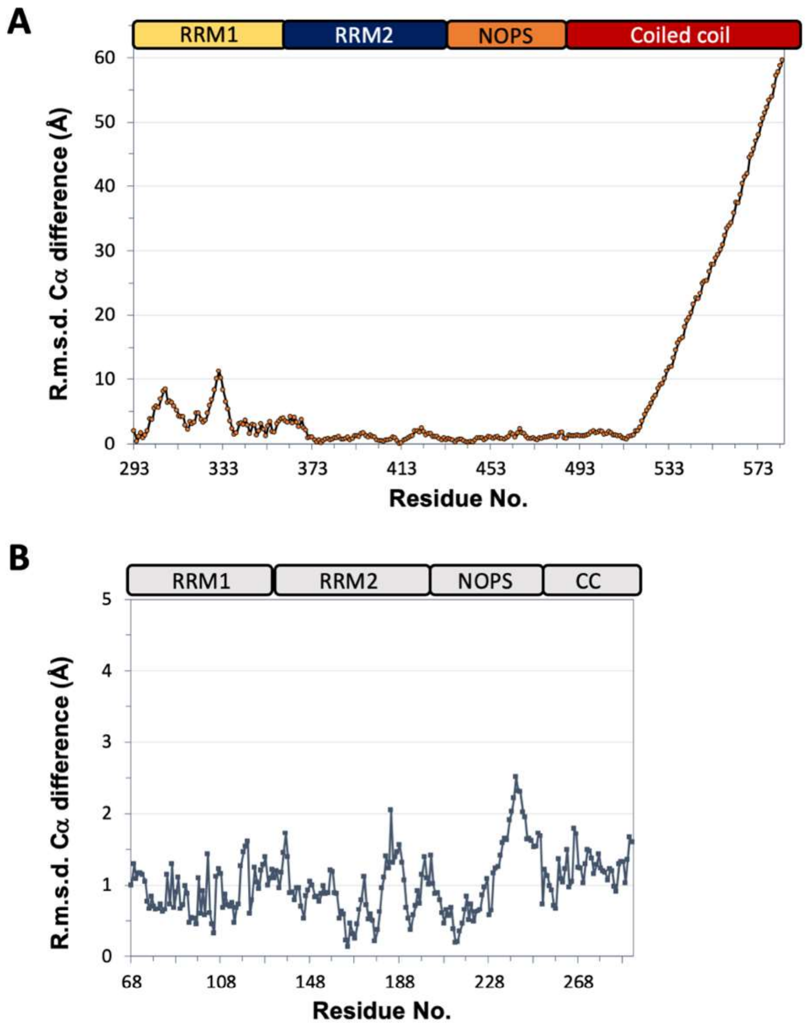

Supplementary Figure 2: **R.m.s.d. Cα difference between the two SFPQ/NONO heterodimers in the asymmetric unit.** *Superposition of the two SFPQ copies shows that the major differences are localized in RRM1 and the distal CC domain (A) while no significant r.m.s.d. differences are observed in NONO (B).*

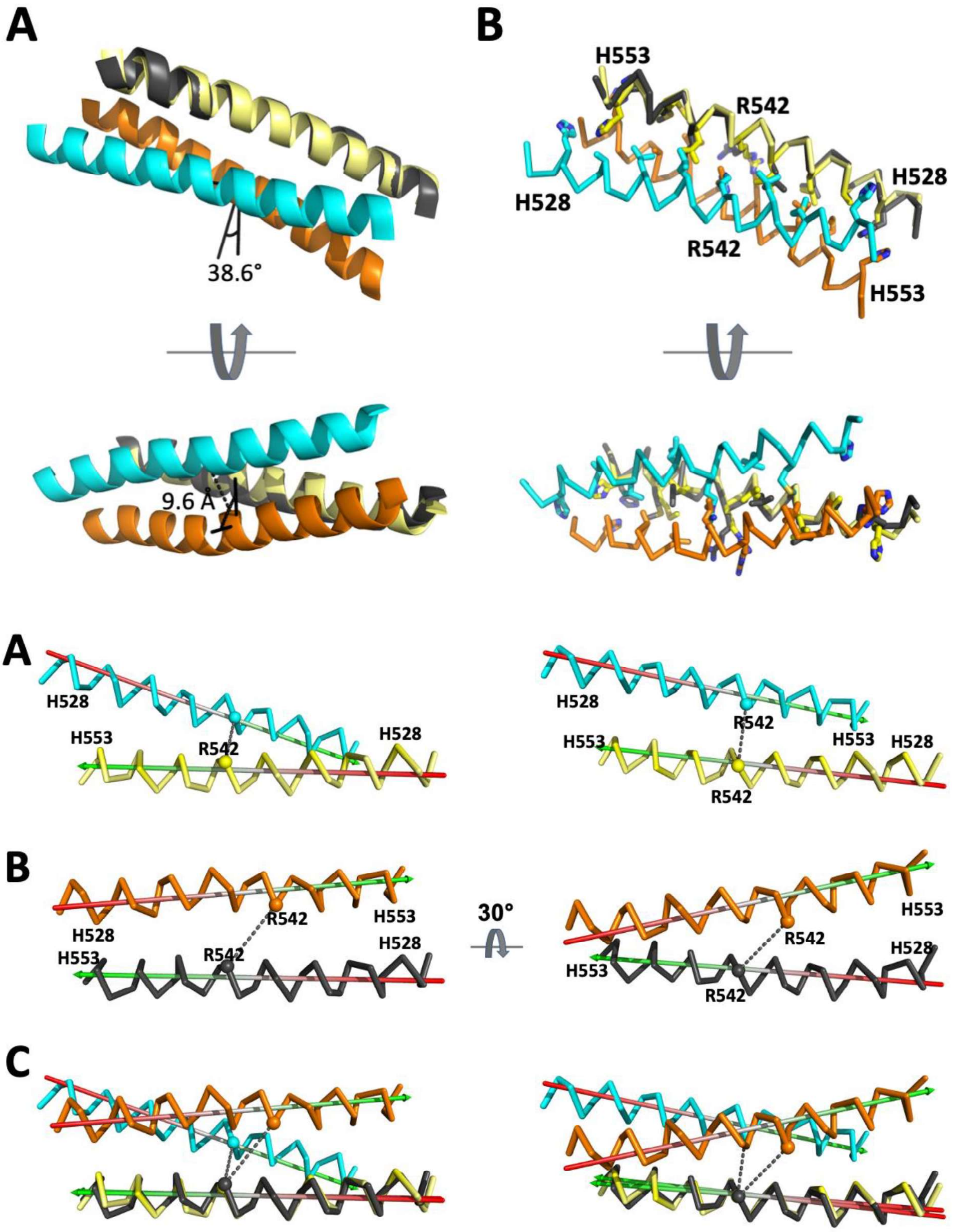

Supplementary Figure 3: **Molecular comparison of the coiled coil interaction motif across different structures**. *The coiled-coil interaction motif (residues 528-555) interface in the SFPQ/NONO structure (Panel A: Chain A (SFPQ) in yellow, Chain C (SFPQ) in cyan; PDB*

entry 6WMZ) and in the SFPQ homodimer (Panel B; Chain A in dark grey, symmetry-related Chain B in orange; PDB entry 4WIJ). Cα atom of R542 is shown in sphere and the distance between the Cα-Cα of R542 in dashed line for comparison purpose. (C) The superposition of Panels A and C.

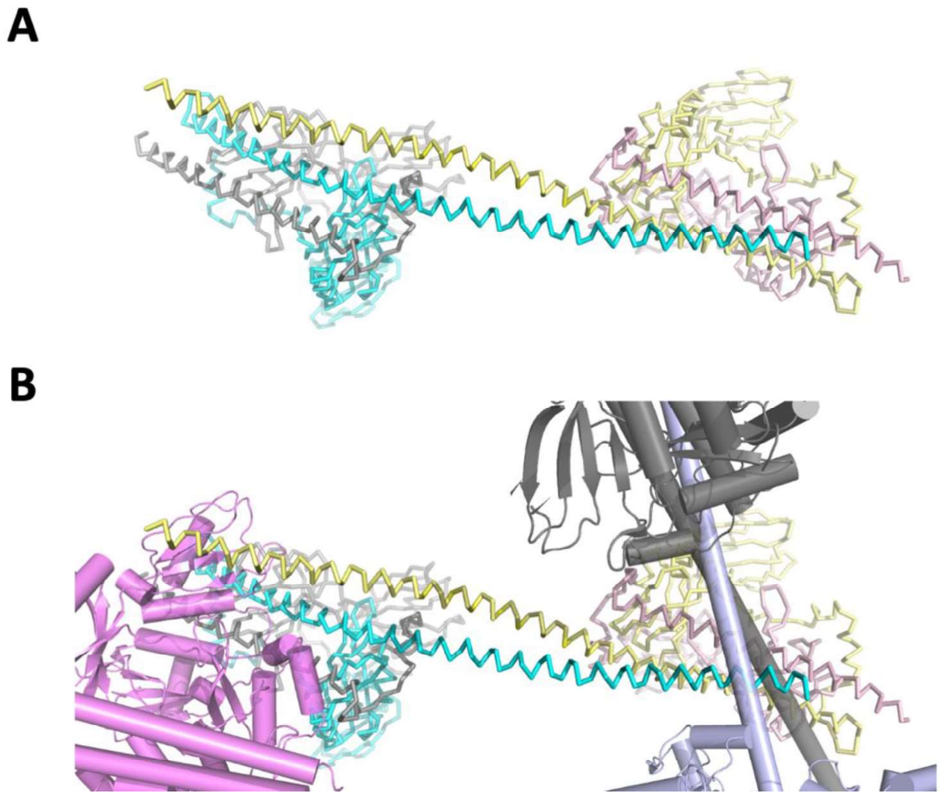

Supplementary Figure 4: **Crystal packing in the distal ends of the CC.** *(A) SFPQ/NONO tetramer formed by the antiparallel coiled-coil interaction in ribbon presentation.(B) Symmetry-related neighboring molecules shown in cartoon presentation (Symmetry operator x-3/2, -y-1/2, -z in magenta; x-1/2, -y-1/2, -z in dark grey; -z-3/2, -y, z+1/2 in light purple).*

Supplementary Table 1. Comparison of the Cα-Cα distances in the coiled-coil interaction motif. The residue which shares the shortest Cα-Cα distance is marked bold.

Homodimer (4WIJ)			Heterodimer (6WMZ)		
Chain A	Chain B [a]	Cα-Cα distance (Å)	Chain A	Chain C	Cα-Cα distance (Å)
H528	H553	8.6	H528	H553	10.4
A532	H553	10.9	A532	**H553**	**7.6**
	M549	8.3		M549	8.1
	**L546**	**7.6**		L546	11.2
L535	M549	10.2	L535	**M549**	**8.4**
	**L546**	**7.6**		L546	9.4
L539	L546	9.7	L539	**L546**	**7.2**
	R542	7.4		R542	7.7
	**L539**	**7.6**		L539	11.5
R542	R542	9.3	R542	**R542**	**8.4**
	**L539**	**7.0**		L539	9.7
L546	L539	9.6	L546	**L539**	**6.8**
	**L535**	**7.9**		L535	8.5
	A532	6.5		A532	11.9
M549	L535	11.1	M549	**L535**	**9.5**
	**A532**	**7.8**		A532	10.7
H553	H528	7.6	H553	H528	8.8

[a] Symmetry operator (y+1/2, -x+1/2, z+1/4)

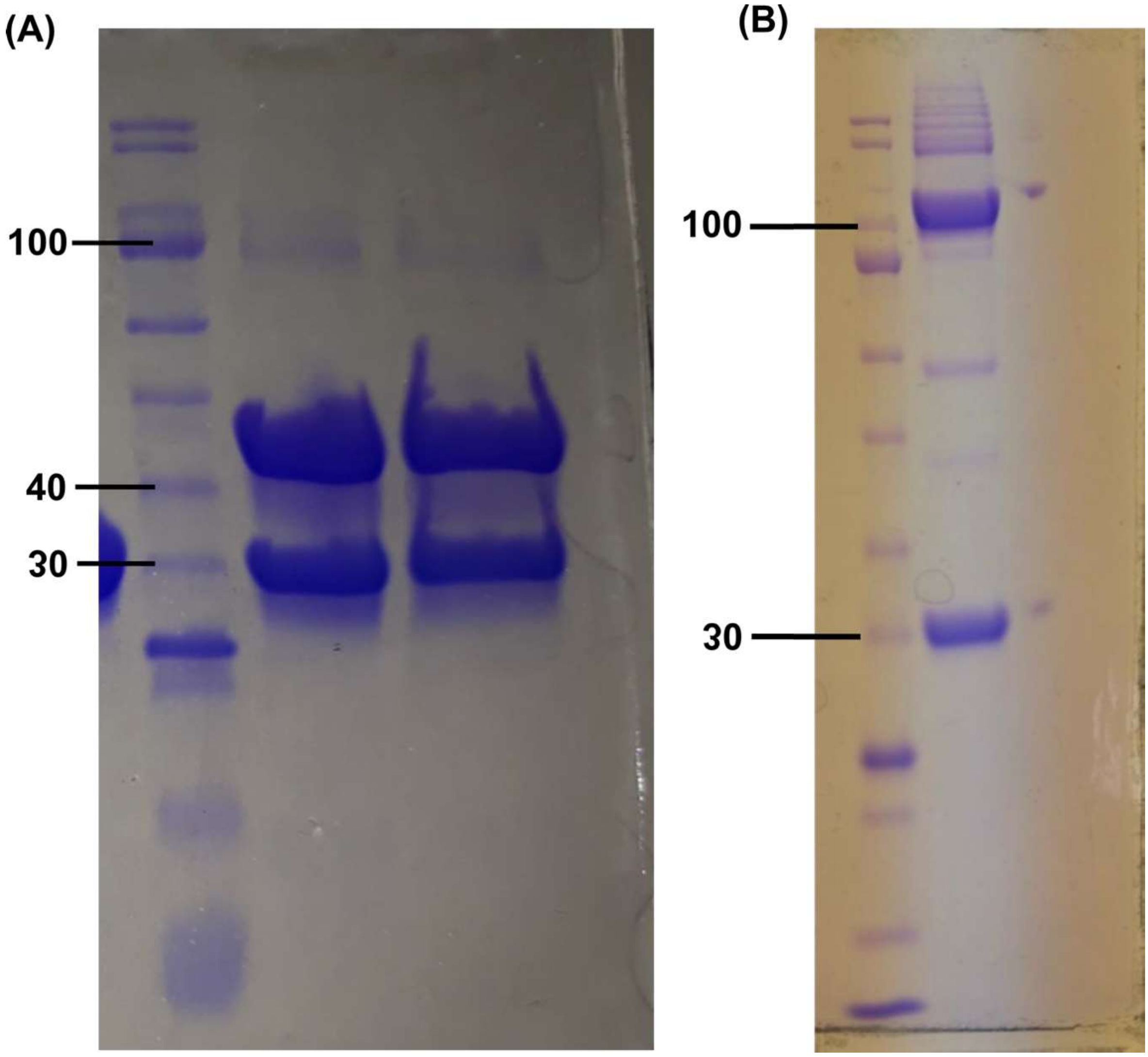

Supplementary figure 5**: SDS-PAGE gel indicating disulfide bond formation in SFPQ R542C**. *(A) Reducing SDS-PAGE with β-mercaptoethanol of the R542C construct (SFPQ214-598(R542C)/NONO53-312). Proteins are running at the expected MW of ~44 kDa for SFPQ and ~30 kDa for NONO (B) Non-reducing SDS-PAGE of the R542C construct (SFPQ214-598(R542C)/NONO53-312). NONO runs at the expected MW of ~30 kDa whilst the band corresponding to SFPQ dimer has shifted to ~100 kDa.*

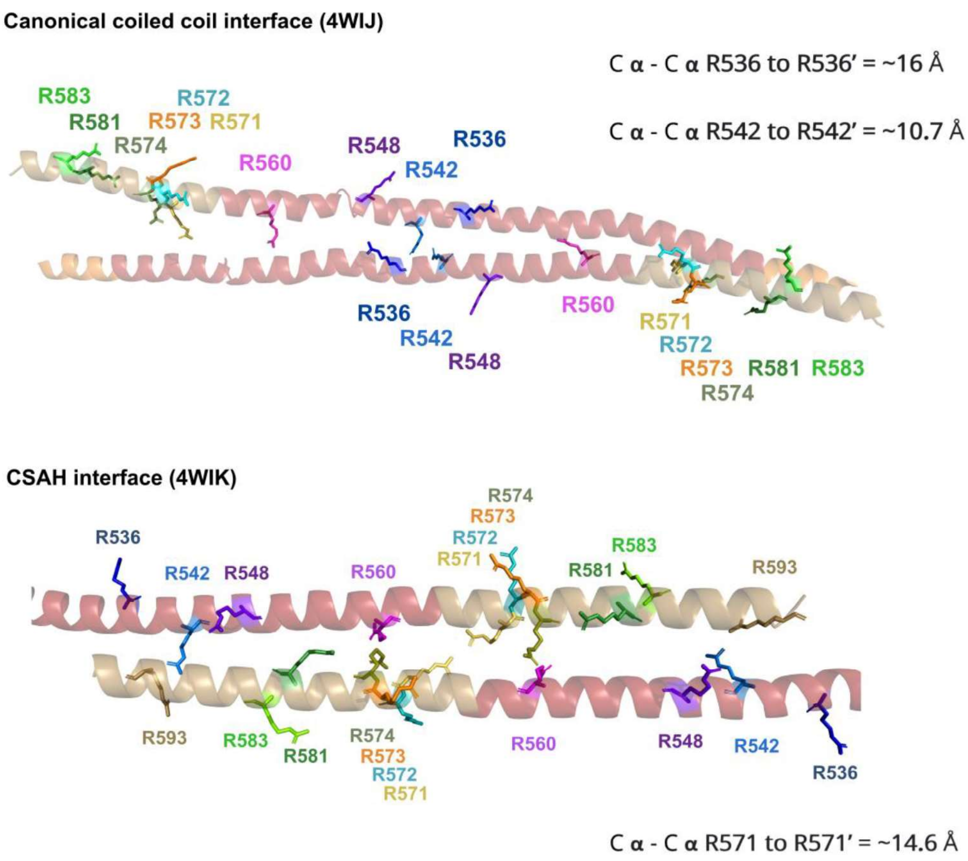

Supplementary figure 6: **A structural schematic indicating some mutation positions retrieved from various databases (see supplementary table 2).** *All positions are coloured and labelled accordingly across the 4WIJ and 4WIK interfaces from Lee et al. (2015). Alpha carbon distances between possible cysteine partners are annotated on the image.*

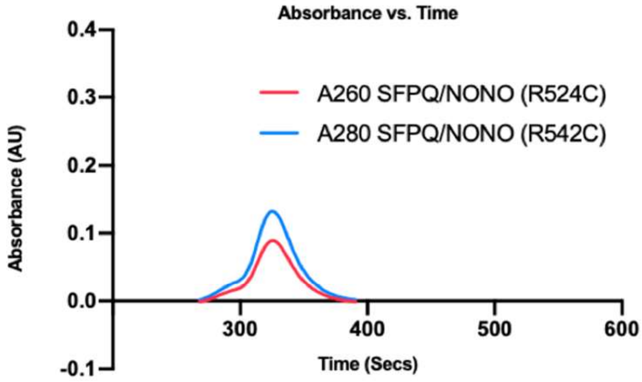

Supplementary figure 7: **SEC-SAXS absorbance chromatogram of SFPQ214-598(R542C)/NONO53-312.** *Absorbance ratios at 260 and 280nm are consistent with that of a pure protein peak.*

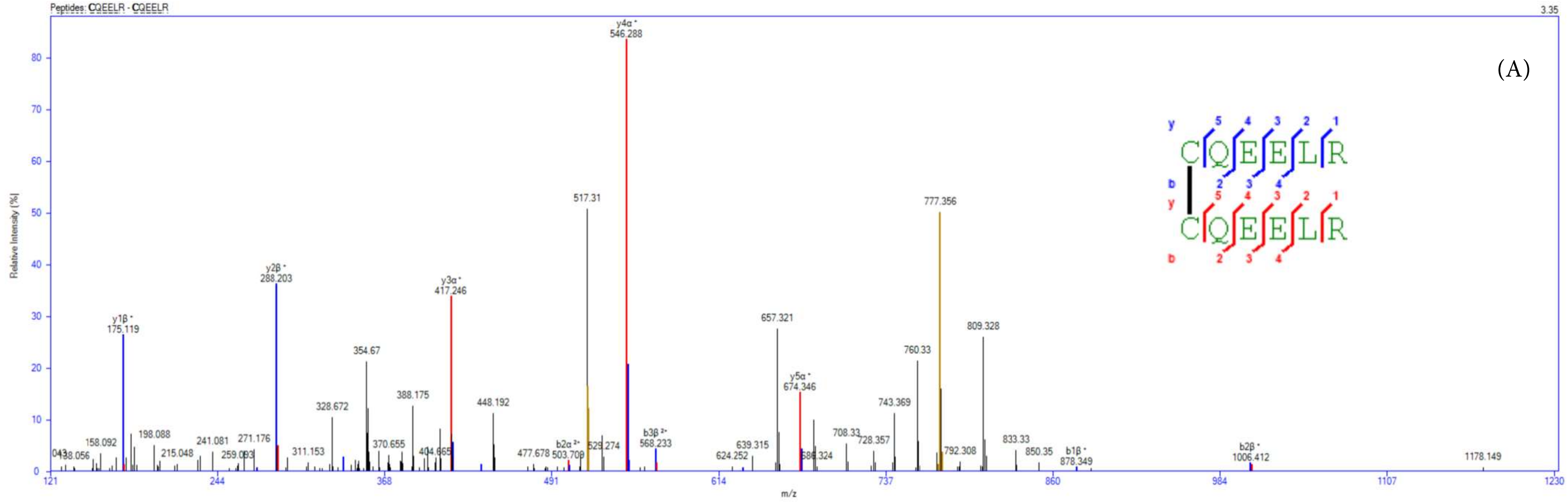
Peptides: CQEELR - CQEELR
3.35
(A)
Relative Intensity [%]
m/z
y1β⁺ 175.119
y2β⁺ 288.203
y3α⁺ 417.246
y4α⁺ 546.288
y5α⁺ 674.346
b2α²⁺ 503.709
b3β²⁺ 568.233
b1β⁺ 878.349
b2β⁺ 1006.412
517.31
777.356
657.321
809.328
760.33
354.67
388.175
328.672
448.192
743.369
CQEELR
CQEELR

(B)

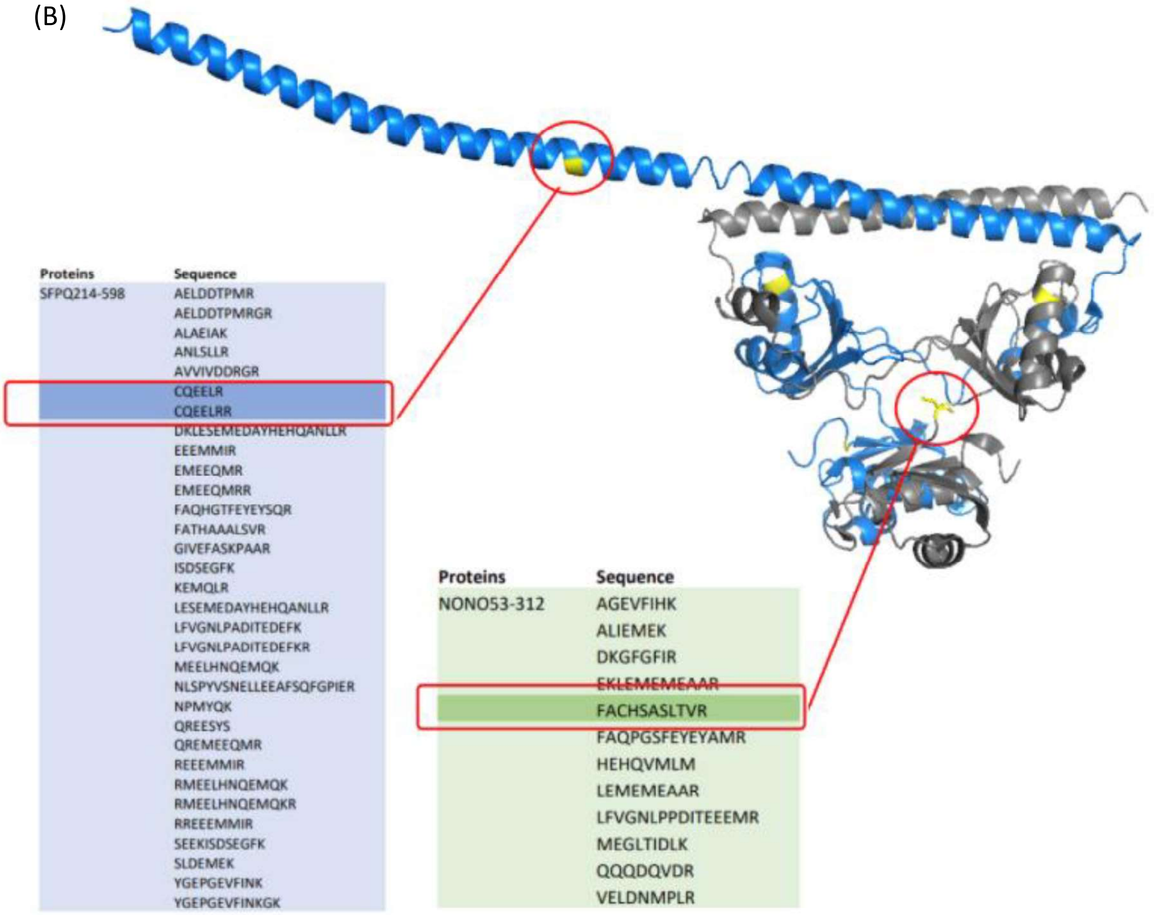

Supplementary figure 8: **Disulfide identification mass spectrometry results for SFPQ R542C.** *(A) MS/MS spectra indicating the presence of a CQEELR-CQEELR disulfide bound peptide. MS/MS spectra of 3H^{+} ion at m/z 517.31 was matched to the peptide CQEELR linked to a second peptide CQEELR with a SS between the two cysteines. (B) Sequence coverage indicating that detected peptides covered 51.3% and 60.4% of NONO and SFPQ respectively. The identification of the FACHSASLTVR peptide in isolation, rather than as part of a disulfide-bonded pair, does not discount its presence. Their abundance could fall below the detection threshold. The disulfide bond linking CQEELR and CQEELR emerges as the predominant factor.*
